# A DNA Virus of *Drosophila*

**DOI:** 10.1371/journal.pone.0026564

**Published:** 2011-10-28

**Authors:** Robert L. Unckless

**Affiliations:** Department of Biology, University of Rochester, Rochester, New York, United States of America; American Museum of Natural History, United States of America

## Abstract

Little is known about the viruses infecting most species. Even in groups as well-studied as *Drosophila*, only a handful of viruses have been well-characterized. A viral metagenomic approach was used to explore viral diversity in 83 wild-caught *Drosophila innubila*, a mushroom feeding member of the quinaria group. A single fly that was injected with, and died from, Drosophila C Virus (DCV) was added to the sample as a control. Two-thirds of reads in the infected sample had DCV as the best BLAST hit, suggesting that the protocol developed is highly sensitive. In addition to the DCV hits, several sequences had *Oryctes rhinoceros* Nudivirus, a double-stranded DNA virus, as a best BLAST hit. The virus associated with these sequences was termed *Drosophila innubila* Nudivirus (DiNV). PCR screens of natural populations showed that DiNV was both common and widespread taxonomically and geographically. Electron microscopy confirms the presence of virions in fly fecal material similar in structure to other described Nudiviruses. In 2 species, *D. innubila* and *D. falleni*, the virus is associated with a severe (∼80–90%) loss of fecundity and significantly decreased lifespan.

## Introduction

The advent of high-throughput DNA sequencing technology has facilitated the discovery and identification of microbes from environmental samples. Though most of the focus has been on metagenomics of microbial communities, leading to the detection of a huge diversity of bacteria and their related bacteriophages [1], [2], [3], viral metagenomic approaches have recently been used to explore viral diversity within individuals exhibiting symptoms ranging from obesity in humans to colony collapse disorder in honey bees to Shaking Mink Syndrome in mink [4], [5], [6], [7].

There is growing appreciation for the important role of interactions among symbionts in host ecology and evolution [8], [9], [10], [11], [12]. In particular, the interaction between vertically and horizontally transmitted microbes and pathogens is the focus of much theoretical and empirical attention [13], [14], [15], [16], [17]. *Wolbachia*, probably the most common vertically transmitted endosymbiont among insects [18], [19], has recently been found to confer resistance to certain RNA viruses in some hosts [14], [15], [20], [21]. However, the importance of such virus protection in natural populations of *Drosophila* has not yet been explored. To investigate the relationship between insect hosts, endosymbiotic bacteria, and viruses, wild-caught *Drosophila innubila* females, about 1/3 of which are infected with *Wolbachia* [22], were screened for virus infection using a viral metagenomic approach.

*D. innubila* is a member of the mushroom-feeding quinaria group of *Drosophila*. They inhabit woodlands and forests of the Sky Islands of Mexico, Arizona, and New Mexico. Adults feed, mate and oviposit on mushrooms and larvae burrow through mushroom tissue, feeding on it prior to pupation. Species in the quinaria group are hosts to endosymbionts such as *Wolbachia* and *Spiroplasma* [23], [24], parasitic nematodes [25], parasitoids [26] and mites (Emma Dietrich, personal communication). As for the vast majority of *Drosophila* species, very little is known about virus infection in natural populations.

Of the roughly dozen different virus identified in *Drosophila* [27], [28], the most well-studied in natural populations is probably *Drosophila* Sigma Virus. Several studies have examined the frequency of *Drosophila* Sigma Virus infection in natural populations, indicating some degree of host specificity and infection frequencies ranging form absence to more than 70% [29], [30], [31]. Some work has also been done on host range of *Drosophila* C Virus, which infects several species from across the genus, with little host specificity [32]. However, many of these lines were maintained in the lab for several generations or from stock centers.

Reported here is the development and implementation of a new virus discovery protocol for *Drosophila* and other insects. This protocol revealed the presence of a new DNA virus that is both taxonomically and geographically widespread and is associated with significant mortality in at least two species of *Drosophila*. *Wolbachia*, however, appears to play no role in protection of *D. innubila* from the adverse effects of the virus.

## Methods

### Samples

Flies for the metagenomic survey were collected in the Chiricahua Mountains, as described in Unckless *et al.* [22], by sweep netting over mushroom baits in 2006 and 2007 near the Southwest Research Station, Portal, AZ. They were then shipped to the lab in Rochester, NY, where females were placed individually in vials and allowed to lay eggs for 6 days. These females were then dissected, their ovaries removed and screened for *Wolbachia*, and the rest of the carcass frozen at −80°C [22]. Forty-two *Wolbachia*-infected and forty-one uninfected carcasses, spanning 2 collection years, were selected for viral screening. Flies that produced few or no offspring were overrepresented, in order to increase the chance of including virus-infected flies. One fly that was injected with, and later died from, Drosophila C Virus (DCV) was added to the *Wolbachia*-infected sample to assess the efficiency of enrichment for viruses.

### Virus enrichment and extraction

A protocol was developed to remove as much host nucleic acid as possible, leaving capsid-protected viral nucleic acids intact before extraction, production of cDNA libraries, and sequencing (Figure S1). The protocol is a modification of several previously published protocols [4], [33], [34]. All flies for each sample were homogenized in 200 µl viral buffer [33], then centrifuged for 5 min. at RT at 2500× g. The supernatant was then transferred to a new centrifuge tube. Genomic DNA was digested by adding 0.1 volumes of DNase I and reaction buffer (AMPD1-1KT, Sigma-Aldrich, USA) and incubating for 15 min. at RT. Sigma stop solution (included in DNase I kit) was added at 0.1 volume, and the solution was incubated at 70°C for 10 min. Genomic RNA was digested with 2 µl 0.02 mg/ml RNase A/T1 (ENN051, Fermentas, Glen Burnie, MD) incubated at 37°C for 3 hours. 1 µl Ribolock (EO0381, Fermentas, Glen Burnie, MD) was added to protect viral nucleic acids. Samples were enriched for viruses because, in any infected fly, the total RNA from a virus will be only a small fraction of the total RNA from the entire fly. In addition, because most flies are probably uninfected with viruses, pooling the flies for virus detection requires selective removal of nucleic acids of the host and resident microbes.

RNA was extracted using the E.Z.N.A. viral RNA extraction kit (R6874-01, Omega Bio-Tek, USA), which will also isolate DNA. Further sample preparation including library preparation (Rapid Library Preparation Method, Roche, Germany), nebulization and emulsion was performed at the Engencore sequencing facility (Columbia, SC). The 2 samples were bar-coded and run on 1/4 of a chip using a Roche 454 machine with Titanium chemistry.

### Data analysis

Both raw reads and contigs (assembled at Engencore using the Roche/454 Life Sciences Newbler algorithm) were analyzed. All searches were performed locally using stand-alone BLAST+ [35] with a minimum E-value of 0.0001. Initially, each contig and read were searched against the RefSeq protein [36] database using BLASTx with the BLOSUM62 matrix and gap costs of 11 and 1 for opening and extension, respectively. Since the goal was in finding viruses, several additional searches were performed with restricted databases to enhance the sensitivity of the search. The restricted databases included all viruses in Viral RefSeq protein [36], 3 RNA virus databases (single-stranded RNA viruses, double-stranded RNA viruses and Drosophila C Virus), and 3 DNA virus databases (Baculoviruses, Nudiviruses, and *Oryctes rhinoceros* Nudivirus). All but the Viral RefSeq protein database were constructed de novo using NCBI's taxonomy browser. The Baculovirus, Nudivirus and *Oryctes rhinoceros* Nudivirus searches were added after identification of a putative Nudivirus in the initial searches (see below). Limiting the size of the database decreases the E-value of any particular match, increasing its significance, because the probability of a chance match decreases. For all searches, significant hits were characterized by parsing the BLAST output and accessing Genbank to identify genes and organisms for the hit. These scripts were written in PERL and utilized functions in BIOPERL [37]. Sequences with BLAST hits to Nudiviruses were deposited in Genbank, except those sequences shorter than 200 bp, which are presented in the online supplemental material (Material S1).

### Survey of wild flies for DiNV infection

After discovery of a putative DNA virus (see results), several species of *Drosophila* were surveyed for infection with this virus. Flies were collected from Rochester, NY and the Southwest Research Station in 2009 and 2010. DNA from 7 *Drosophila phalerata* (4 females and 3 males) individuals collected in Munich, Germany was kindly provided by Kelly A. Dyer. In addition, *D. innubila* were collected in 2010 at the Southwest Research Station and immediately dissected and extracted on site to minimize possible horizontal transmission of the virus among flies. DNA was extracted using the Puregene DNA purification kit (QIAGEN, Valencia, CA). Flies were screened for the virus using standard insect *COI* primers (1490 and 2198) [38] as a control for extraction quality and newly developed primers (P47F: 5′–TGAAACCAGAATGACATATATAACGC and P47R: 5′–TCGGTTTCTCAATTAACTTGATAGC) for the *P47* homolog found in the metagenomics search. For each species, the *P47* locus from at least one individual was sequenced using BigDye Terminator v3.1 (#4337455, Applied Biosystems, Carlsbad, CA) and deposited in GenBank (Accession numbers JN44311–JN44330). These sequences were used to build a phylogenetic tree using PhyML [39] with the HKY85 model of substitution and 100 bootstrap replicates and Mr. Bayes [40] with the GTR+Gamma model with a chain length of 1,100,000 and a burn in of 100,000 generations. The *P47* ortholog from OrNV was used as an outgroup.

### Electron microscopy of virus particles

Because transmission of the DNA virus may be fecal-oral, as hypothesized for the closely related *Oryctes rhinoceros* Nudivirus [41], fly fecal material was scraped from the side of vials containing infected *D. falleni* and then PCR screened for the virus, using the methods described above. These samples were almost invariably positive for the virus, so fecal material was primarily used for imaging. To concentrate the virus on a microscope slide, a crowded vial of infected flies was inverted on a microscope slide and flies were allowed to defecate for 4 d. A small sample was scraped and PCR screened for the virus. An attempt was made to find virus particles in whole flies by dissecting out the digestive tract for imaging.

The glass slide with deposited feces was fixed in 0.1 M sodium cacodylate buffered 2.5% glutaraldehyde for 24 hours and post-fixed in 1.0% buffered osmium tetroxide for 20 min. The slide was transitioned through a graded series of ethanol to 100% (×3) and infiltrated with Spurr epoxy resin overnight. The next day, size 3 BEEM capsules were filled with fresh resin and inverted and placed onto the glass slide over the fecal matter. The slide was placed into a 600°C oven and allowed to polymerize overnight. The polymerized BEEM capsules were removed from the glass slide using the “pop-off” technique [42] which involves dipping several times into liquid nitrogen. The capsules containing the entrapped fecal material were trimmed with a razor blade to a small trapezoid and thin sectioned on a Reichert ultramicrotome using a diamond knife at 70 nm. The sections were placed onto 200 mesh copper grids and stained with aqueous uranyl acetate and lead citrate. The grids were examined using a Hitachi 7650 Transmission Electron Microscope and micrographs were captured using an attached Gatan Erlangshen 11 megapixel digital camera.

### Fitness of infected flies

Wild-caught females were used to assess the survival and fecundity of flies as a function of infection with virus and *Wolbachia*. In September 2009, flies were captured near the Southwest Research Station as described above and transferred to sugar agar for transport to Rochester, NY, which took ∼6 d. In Rochester, flies were placed on mushroom food (instant drosophila medium plus a piece of commercial *Agaricus bisporus* mushroom and a cotton roll) for egg laying. Females were then moved to new vials every other day until they died. The experiment lasted for 55 days, at which time no flies were laying fertilized eggs. Females were screened for the DNA virus and their offspring were counted as they emerged. In July and August 2010, the same protocol was followed for *D. falleni*. In this case, flies were collected around Rochester, NY and were established in culture the day they were collected, but the experiment was terminated after 10 days. As described above, flies from the 2010 *D. innubila* collection were dissected on site and mature eggs (stage 10 or later) in both ovaries were counted to assess fitness costs associated with the virus. All flies were PCR screened for the DNA virus and, as a control, insect *COI*.

### Experimental infection of lab-reared flies

To directly assess the fitness consequences of viral infection, flies were injected with live virus and survival was monitored. Since cell culture for this virus has not yet been established, live virus was isolated as follows. Several wild *D. innubila* females were homogenized individually in viral buffer (see above) and centrifuged at low speed for one minute to remove large fly debris. The supernatant was then spun through a 0.45 µm filter (UFC30HV25, Millipore USA, Billerica, MA). 10 µl of the filtrate for each sample was used to PCR screen for the virus, using the DNA extraction and PCR protocols as described above. The filtrate from 4 flies identified by PCR as positive for DiNV infection were pooled as the virus-positive, and 4 flies screening negative for DiNV were pooled as the virus-negative control. Each sample was diluted 1∶10 before being used for injection. A separate control, sterile virus buffer was also employed. Six- to eight-day-old male and female *D. innubila* and *D. falleni* were injected with about 200 nL of one of the three treatments using a Narishige IM300 microinjector (Narishige, Japan). Fly survival was monitored daily and flies were kept at low density (maximum = 10 per vial) and transferred to new food every other day until most flies injected with the virus-positive filtrate were dead. Both the virus-positive and virus-negative samples were examined using electron microscopy to be sure a) only DiNV was present in the virus-positive samples and b) no bacteria were present in either sample.

### Vertical transmission

For a subset of wild-caught *D. innubila* mothers found to be infected with the DNA virus, offspring were PCR screened to assess vertical transmission of the virus. Offspring were frozen 2–6 d after emergence, and DNA was later extracted and screened as described above. A total of 27 daughters and 2 sons of were screened from 10 virus-infected females. The low offspring production of virus-infected females limited the sample size and male-killing by *Wolbachia* resulted in the highly skewed sex-ratio.

### Permits for fly collections

All necessary permits were obtained for the described field studies. Since it involved protected lands, flies collected in the Chiricahua Mountains were collected with permission of the National Forest Service (Drosophila innubila in the Coronado National Forest System; August 2006 through October 2009; Authorization ID SUP0040-01). Collections in the Chiricahua Mountains in 2010 were conducted on the grounds of the Southwest Research Station with permission from the station director, Dawn Wilson. Permits were not required for collections around Rochester, NY and all collections were performed on the property of the author and principal investigator, John Jaenike. Flies collected in Germany were processed and sent as DNA by a third party. No species of *Drosophila* are listed as threatened or endangered.

## Results

### Viral metagenomics

A total of 1225 raw reads and 25 contigs (using 1154 raw reads) were recovered for the *Wolbachia* uninfected sample and 44,675 raw reads and 124 contigs (using 5939 raw reads) were recovered for the *Wolbachia* infected sample. Raw reads averaged 320.3 bp for the uninfected sample and 394.9 bp for the infected sample.

Table 1 shows the types of hits for each search and sample. An E-value cutoff of 0.0001 was used for all searches. Hits to bacterial and eukaryotic sequences were overwhelmingly to ribosomal RNA genes (data not shown), which could be due to the high ratio of ribosomal RNA transcripts to gene-specific mRNA transcripts and/or because the ribosome somehow protects ribosomal RNA from RNase digestion.

**Table 1:** Summary of BLAST hits from viral metagenomic survey.

Sample	Database	Reads	Hits	Euk	Bact	Viruses	Dipt	Other Arth	Mammal/Plant/Fungi	Nud	Bac	RNA virus*	DNA virus**	DCV	OrNV
W− raw	Refseq	1225	930	773	119	37	71	311	43/21/8	26	0	0	2	9	26
W+ raw	Refseq	44675	40124	10477	2586	27012	912	3422	964/205/132	3	0	318	2	26688	3
W− contigs	Refseq	25	21	8	11	2	2	1	0/0/1	2	0	0	0	0	2
W+ contigs	Refseq	124	113	17	51	29	1	4	2/0/0	0	0	0	0	29	0
W− raw	V. Refseq	1225	41	NA	NA	41	NA	NA	NA	26	2	0	6	9	26
W+ raw	V. Refseq	44675	27216	NA	NA	27216	NA	NA	NA	3	0	321	15	26874	3
W− contigs	V. Refseq	25	2	NA	NA	2	NA	NA	NA	2	0	0	0	0	2
W+ contigs	V. Refseq	124	33	NA	NA	33	NA	NA	NA	0	0	1	0	32	0

Abbreviations: V. Refseq = viral Refseq database; Euk = Eukaryotes; Bact = Bacteria; Dipt = Diptera; Other Arth = Other Arthropods; Nud = Nudiviruses; Bac = Baculoviruses; DCV = Drosophila C Virus; OrNV = Oryctes rhinoceros Virus.

*RNA viruses other than DCV.

**DNA viruses other than OrNV.

The *Wolbachia*-infected sample was spiked with 1 fly that had been injected with Drosophila C virus (DCV). Two-thirds (26,688) of reads with significant hits in the infected group had DCV as the best hit, as opposed to less than 1% (9 reads) in the uninfected group, demonstrating both the sensitivity and specificity of the method. Figure S2 shows the distribution of hits across the DCV genome and simple calculations show that the average coverage is more than 1200×.

All virus hits are summarized in Table 2. There were 27 unique virus hits, including 9 bacteriophage, and 12 double-stranded DNA, 3 single-stranded RNA, and 3 double-stranded RNA viruses. Sixteen virus families were represented, whose normal hosts include a variety of organisms.

**Table 2:** Summary of BLAST hits to viruses.

Virus Species	Count	Virus Type	Virus Family	Host
Acanthamoeba polyphaga mimivirus	1	dsDNA	Mimiviridae	Ameoba
Avian leukosis virus	1	ssRNA(RT)	Retroviridae	Birds
Bathycoccus sp. RCC1105 virus	1	dsDNA	Phycodnaviridae	Green algae
Cauliflower mosaic virus	1	dsDNA(RT)	Caulimoviridae	Brassicaceae
Cricket paralysis virus	312	ssRNA(+)	Dicistroviridae	Arthropods
Dioscorea bacilliform virus	1	dsDNA(RT)	Caulimoviridae	Dioscoreaceae
Drosophila C virus	26915	ssRNA(+)	Dicistroviridae	Arthropods
Enterobacteria phage FI sensu lato	2	ssRNA(+)	Leviviridae	Bacteria
Enterobacteria phage Qbeta	3	ssRNA(+)	Leviviridae	Bacteria
Enterobacteria phage WV8	1	dsDNA	Caudovirales	Bacteria
Great Island virus	3	dsRNA	Reoviridae	Birds*
Heliothis armigera cypovirus 5	1	dsRNA	Reoviridae	Arthropods
Klebsiella phage phiKO2	1	dsDNA	Siphoviridae	Bacteria
Lymantria dispar MNPV	1	dsDNA	Baculoviridae	Arthropods
Lymphocystis disease virus	1	dsDNA	Iridoviridae	Fish
Mycobacterium phage TM4	1	dsDNA	Siphoviridae	Bacteria
Oryctes rhinoceros virus	31	dsDNA	Nudivirus†	Arthropods
Ostreococcus lucimarinus virus	1	dsDNA	Phycodnaviridae	Green algae
Paramecium bursaria Chlorella virus 1	3	dsDNA	Phycodnaviridae	Green algae
Peruvian horse sickness virus	1	dsRNA	Reoviridae	Vertebrates*
Phthorimaea operculella granulovirus	1	dsDNA	Baculoviridae	Arthropods
Prochlorococcus phage P-SSM2	2	dsDNA	Myoviridae	Bacteria
Prochlorococcus phage P-SSM4	1	dsDNA	Myoviridae	Bacteria
Pseudomonas phage PA11	1	dsDNA	Unclassified	Bacteria
Shrimp white spot syndrome virus	2	dsDNA	Nimaviridae	Arthropods
Synechococcus phage S-RSM4	2	dsDNA	Myoviridae	Bacteria
Trichoplusia ni ascovirus 2c	1	dsDNA	ascoviridae	Arthropods

*arthropod vectored;

† no family name.

Three viruses in the list stand out. First was DCV, which was expected to be present in large quantities. The second most common virus hit was to Cricket paralysis virus (CrPV), which is closely related to DCV and therefore may represent poor quality reads that were actually DCV. However, CrPV has a broad host range and can infect *Drosophila melanogaster* [43], [44], so these reads may represent a real RNA virus, closely related to CrPV, that infects *Drosophila innubila*.

Finally, 29 reads (26 and 3 from the *Wolbachia*-uninfected and *Wolbachia*–infected samples, respectively) had *Oryctes rhinoceros* Nudivirus (OrNV), a double-stranded DNA virus, as the best hit. Narrowing the search database to just viruses did not increase the number of sequences hitting OrNV, but searches restricted to Nudiviruses increased this to 31 and 7 (see Table 3) from the *Wolbachia*-uninfected and infected samples, respectively, and searching against OrNV itself increased it to 30 and 3.

**Table 3:** Amino acid sequence similarity between *Drosophila innubila Nudivirus* sequences and other Nudiviruses.

Read	bp	Accession	OrNV best hit (Accession)	OrNV AA% ID (length)	GbNV best hit	GbNV AA %ID (length)	HzNV1 best hit	HzNV1 AA %ID (length)
8IZQUB	125	NA*	polh/gran (YP_002321327)	46% (37)				
8JTQNZ	158	NA*	vp91 (YP_002321417)	52% (51)	VP91 capsid protein (YP_001111269)	44% (49)	p91 capsid protein (NP_690465)	28% (51)
8JQMLB	174	NA*	gp78-like protein (YP_002321338)	53% (37)	hypothetical protein GrBNV_gp78 (YP_001111345)	40% (58)		
ConA	194	NA*	gp72-like protein (YP_002321333)	58% (53)	hypothetical protein GrBNV_gp72 (YP_001111339)	33% (42)		
7IIS77	314	JN680861	rr1 (YP_002321362)	31% (36)	ribonucleotide reductase large subunit (YP_001111349)	45% (31)	Rr1 (NP_690514)	31% (32)
8JFHBN	415	JN680862	pif-2 (YP_002321328)	60% (134)	per os infectivity factor 2 (YP_001111333)	52% (135)	Orf123 (NP_690542)	33% (123)
7H8E3L	426	JN680863					Rr1 (NP_690514)	34% (107)
7H5KKI	453	JN680864	gp97-like protein (YP_002321355)	68% (91)	hypothetical protein GrBNV_gp97 (YP_001111364)	33% (84)		
8I1EHP	481	JN680865	vp39 (YP_002321326)	49% (84)	hypothetical protein GrBNV_gp64 (YP_001111331)	33% (81)		
ConB	521	JN680866	dnahel (YP_002321345)	41% (68)				
ConC	662	JN680867	gp67-like protein (YP_002321329)	66% (41)	hypothetical protein GrBNV_gp67 (YP_001111334)	50% (42)		
ConD	757	JN680868	guanylate kinase-like protein (YP_002321334)	34% (164)	putative guanylate kinase (YP_001111341)	31% (112)		
ConE	761	JN680869	odv-e56 (YP_002321426)	26% (144)	occlusion-derived virus envelope-56 protein (YP_001111272)	29% (140)		
ConF	900	JN680870	rr2 (YP_002321413)	58% (264)	ribonucleotide reductase small subunit (YP_001111330)	31% (225)	Rr2 (NP_690492)	20% (191)
ConG	1525	JN680871	P47 (YP_002321331)	51% (335)	hypothetical protein GrBNV_gp69 (YP_001111336)	26% (323)		
			Ac146-like protein (YP_002321330)	56% (81)	hypothetical protein GrBNV_gp68 (YP_001111335)	21% (84)		

*The GFAK6NV0 prefix has been removed from all read names.

ConA is a contig of 8JS03H, 8JH2MO.

ConB is a contig of 7H1TKA, 7HXBFS.

ConC is a contig of 8I7N7M, 8JEGW7.

ConD is a contig of 8I715A, 8I4S21, 8JPIRD, 8I5BT9, 8JI2RY, 8I54ZI.

ConE is a contig of 8JUZHW, 8JL8DP.

ConF is a contig of 8JJ75Z, 8JIXU2.

ConG is a contig of 8JDDM9, 8JN0QY, 8JGLGS, 8JIZXS,8JC5KB, 8I4WED, 8JMNLG, 8I1J6U, 8JH7ES, 8JNLK0.

OrNV is a member of the small and unclassified Nudivirus group [45], [46]. Nudiviruses have large genomes - OrNV is almost 130 kb - and are characterized by a rod-shaped virion. The search against all Nudivirus proteins found 16 unique Nudivirus proteins among the raw reads (Table 3). For 13 of these, the best hit was OrNV, representing about 10% of the 139 predicted proteins [41] for OrNV (Figure S3). A total of 9540 bases hit OrNV yielding a genomic coverage of about 0.07×. Two more sequences hit the closely related dsDNA virus family Baculoviridae and had marginally significant hits to OrNV when searched against only the OrNV genome. In accordance with proposed Nudivirus nomenclature [47], the above virus will be referred to as *Drosophila innubila* Nudivirus (DiNV). Table 3 shows the percent identity from BLAST hits between each DiNV sequence and the other Nudiviruses yielding matches. Because both sequence and gene conservation is so low in the Nudiviruses, a phylogenetic analysis of DiNV within the Nudiviruses is not possible with the current data. Based on the data in Table 3, it appears that DiNV is most closely related to OrNV. The remainder of the paper will focus on DiNV.

### Survey of wild flies

DiNV virus is present in several *Drosophila* species, including members of 3 subgenera, from both northeastern and southwestern United States, but is not found in a small sample of *D. phalerata* from Europe (Table 4). The prevalence of infection varies among species and is most common within members of the quinaria group. If all species are included, males (36%) were infected significantly more often than females (25%; P = 0.005, FET). Within the 2010 collection of *D. innubila*, males were also significantly more likely to be infected than females (P = 0.0037, FET). A phylogenetic tree of *P47* sequences from each species is presented in Figure S3. All isolates from the Arizona collection had identical sequences, while there was some variation in sequences from New York. The sequences from a *Drosophila simulans* male and a *Drosophila melanogaster* or *simulans* female (both from the subgenus Sophophora) were identical, but were nested within sequences from other members of the subgenus Drosophila.

**Table 4:** Frequency of DiNV infection in wild species. (AZ = Portal, AZ; NY = Rochester, NY; DE = Munich, Germany).

Species	Subspecies (group)	Collection Location	Prop. ♀ infected (# screened)	Prop. ♂ infected (# screened)
*D. psuedoobscura*	Sophophora (obscura)	AZ	0.17 (6)	0 (9)
*D. melanogaster* †	Sophophora (melanogaster)	NY	2 (85)	1 (40)
*D. grisea*	Hirtodrosophila	AZ	0 (17)	0.10 (29)
*D. duncani*	Hirtodrosophila	NY	0 (29)	ND
*D. neotestacea*	Drosophila (testacea)	NY	0 (46)	0.06 (35)
*D. macroptera*	Drosophila (macroptera)	AZ	0.5 (4)	ND
*D. munda*	Drosophila (quinaria)	AZ	0.27 (11)	0 (1)
*D. tenebrosa*	Drosophila (quinaria)	AZ	0.55 (60)	0.65 (20)
*D. recens*	Drosophila (quinaria)	NY	0 (22)	ND
*D. falleni*	Drosophila (quinaria)	NY	0.03 (95)	ND
*D. innubila*	Drosophila (quinaria)	AZ	0.41 (148)*0.37 (198)**	ND0.56 (84)**
*D. phalerata*	Drosophila (quinaria)	Germany	0 (4)	0 (3)

† a mix of both *D. melanogaster* and *D. simulans*;

*2009 collection;

**2010 collection;

ND = no data.

### Electron microscopy

**Figure 1:**
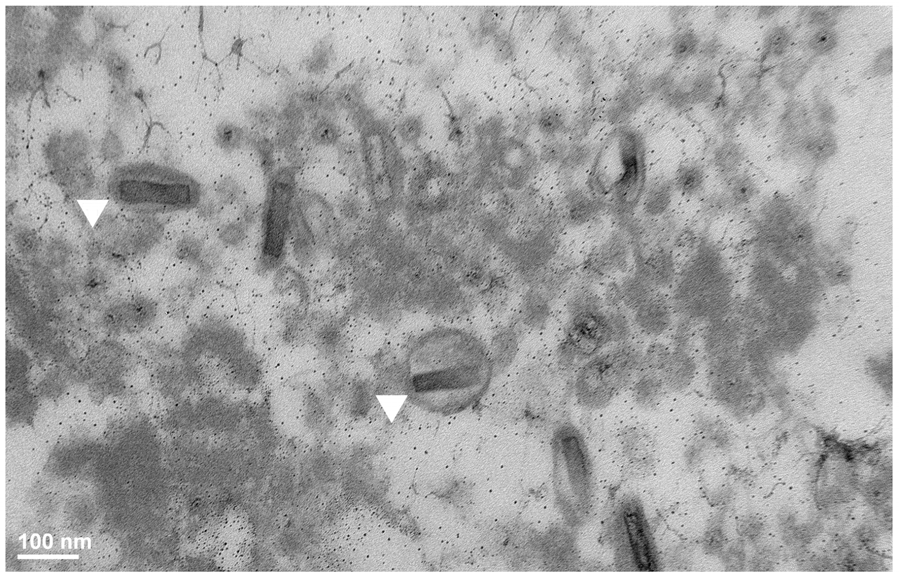
Electron micrograph of *Drosophila innubila Nudivirus* isolated from fecal material of *Drosophila falleni*. Arrowheads point to virus particles.

The fecal material contained numerous virus particles morphologically similar to Baculoviruses and Nudiviruses (Figure 1). The capsid is approximately 120×30 nm, with an envelope ∼135 nm in diameter, making it small among the Nudiviruses [47]. No virus particles were observed from the digestive tracts of flies.

### Fitness effects in wild-caught flies

**Figure 2:**
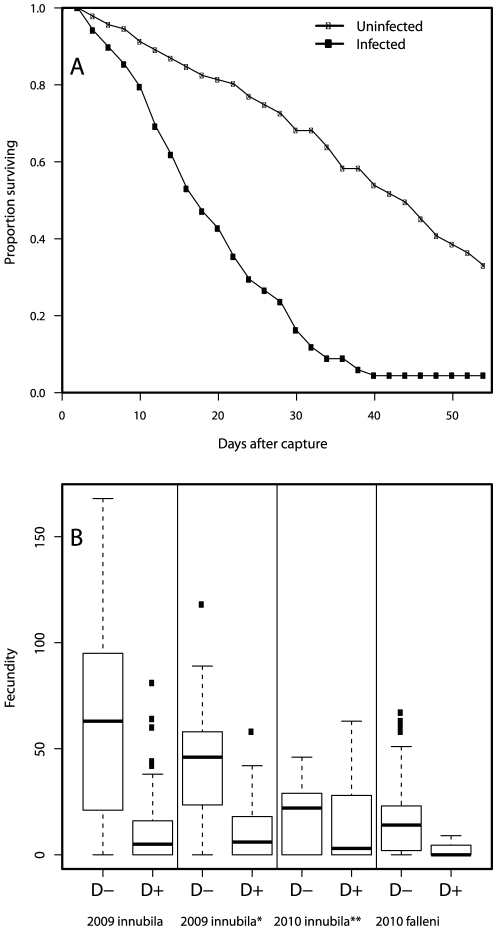
Fitness costs of infection with DiNV. A) Survival of wild-caught *D. innubila* females infected with DiNV or uninfected, diagnosed by PCR; B) actual or potential offspring production by wild-caught females: 2009 *innubila* – lifetime daughters produced; 2009 innubila* - daughters produced in the first 6 d after capture; 2010 innubila** - number of mature eggs in both ovaries; 2010 *falleni* – daughters produced in the first 10 d after capture.

Among female *D. innubila* collected in 2009, those infected with DiNV had significantly decreased lifespan (median survival = 18 and 43 d for virus-infected and uninfected flies, respectively; Kaplan-Meier analysis, P<0.0001; Figure 2A). DiNV-infected females produced ∼80% fewer daughters (mean infected = 11.63; S.E. = 2.03; mean uninfected = 62.96; S.E. = 4.54; t = 10.3; d.f. = 122.87; P<0.0001; Figure 2B), which could result in part from the reduced lifespan of infected flies. Considering only the first 6 days of the experiment and only those flies that survived this period, there was still a highly adverse effect of infection on offspring production (mean infected = 11.26; S.E. = 1.66; mean uninfected = 43.17; S.E. = 2.63; t = 10.27; d.f. = 137.06; P<0.0001). There was no interaction between *Wolbachia* and virus infection on offspring number, suggesting that *Wolbachia* does not protect against the adverse effects of DiNV.

In 2010, the number of mature eggs per ovary was determined in *D. innubila* within 2 hours of capture. The number of mature eggs was significantly less in flies infected with DiNV (mean infected = 14.25; S.E. = 1.86; mean uninfected = 18.54; S.E. = 1.27; t = 1.91; d.f. = 137.34; P = 0.029; Figure 2B).

Only 3 of 95 wild-caught *D. falleni* were infected with DiNV. Because *D. falleni* is not infected with a male-killing bacteria, total offspring were considered instead of only female offspring. DiNV-infected flies produced ∼82% fewer total offspring (mean infected = 3.00; S.E. = 3.00; mean uninfected = 16.57; S.E. = 1.77; t = 3.89; d.f. = 3.63; P = 0.01; Figure 2B).

### Experimental infection of lab-reared flies

**Figure 3:**
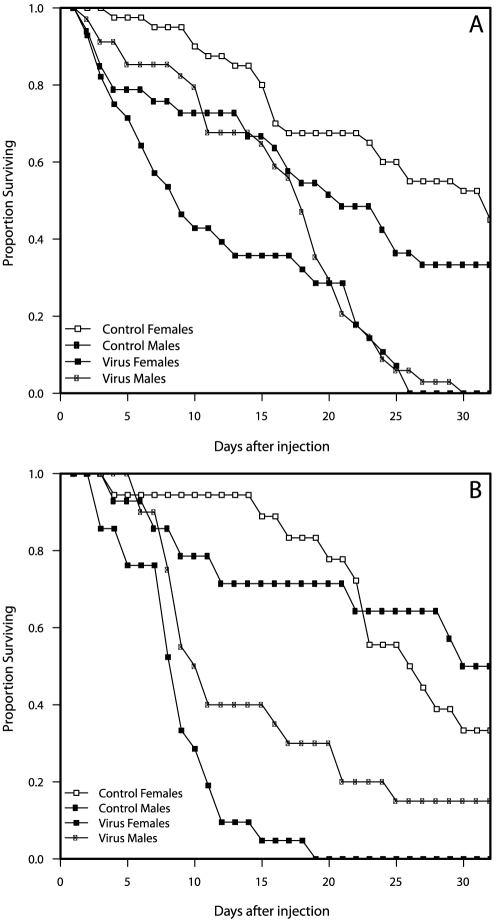
Proportion survival of A) *D. innubila* and B) *D. falleni* after injection with ∼200 nL DiNV-positive filtrate. For clarity, only virus-positive and viral buffer control are presented.

After injection, fly survival was monitored daily until most flies injected with the virus-positive filtrate had died (after 33 days the experiment was stopped, at which point 3 male *D. falleni* injected with the virus were still alive). To assess survival after injection with the three treatments (virus-positive, virus-negative and virus buffer) a Cox proportional hazard model was used with treatment, species and sex as factors. Flies injected with the virus-negative filtrate experienced similar mortality to those injected with virus buffer (P = 0.44), suggesting that nothing in the virus-negative filtrate significantly affected fly mortality. This was further supported by the absence of any recognizable structures (viral, bacterial or otherwise) in the electron micrographs of the virus-negative filtrate. Flies injected with the virus-positive filtrate experienced much higher mortality than those injected with the virus-negative filtrate (P<10^−6^; Figure 3). The median survival time for flies injected with the virus-positive sample was less than that of flies injected with virus-negative filtrate for both males and females of both species (*D. innubila* males: 17 vs. 20 d; *D. innubila* females: 8 vs. 31d; *D. falleni* males: 9.5 vs. 31d; *D. falleni* females: 8 vs 25.5d). There was a moderately significant interaction (P = 0.06) between sex and treatment with males injected with the virus-positive filtrate surviving several days longer than females (see Figure 3). Overall, *D. falleni* experienced higher mortality (P = 0.005) than *D. innubila* regardless of treatment or sex.

### Vertical transmission

Of the 27 female offspring screened for infection only 1 was positive, while neither of the 2 male offspring was positive, for an overall “vertical” transmission rate of 0.034. Note that the single positive offspring may have contracted the virus periorally and therefore, effectively horizontally, making this an upper estimate of vertical transmission.

## Discussion

Using viral metagenomics a putative Nudivirus was discovered to infect several species of *Drosophila*. The virus was termed *Drosophila innubila Nudivirus*. Though the host specificity of the virus is not yet known, the species name was retained for clarity. This may need to be revised later. The abbreviation of *Drosophila innubila Nudivirus* is problematic because conventions and practical concerns in the *Drosophila* community are at odds with those in the Nudivirus community. For example, with more than 2000 species of *Drosophila*, species abbreviations usually employ the first three letters of the species name, Dinn for *Drosophila innubila*. In addition to the Nudivirus described here, *Drosophila* is host to Nora Viruses and Noda Viruses, so using NV could be confusing. For those working on *Drosophila*, DinnNuV might be most informative, although quite cumbersome. Following Nudivirus conventions, the abbreviation would be DiNV. For the current manuscript, the simpler DiNV will be used. While several RNA viruses are well-characterized from *D. melanogaster* and its close relatives [27], [28], DiNV is the first report of a DNA virus in *Drosophila*. As *Drosophila* are an important model for the study of the molecular biology and evolution of immunity, this discovery broadens the scope of host-pathogen interactions that can be studied in the genus.

### Drosophila innubila Nudivirus

DiNV is similar in sequence to the double-stranded DNA viruses of the Nudivirus group, being most closely related to OrNV, which infects rhinoceros beetles. Supporting the conclusion that the virus discovered in *D. innubila* is a Nudivirus, electron microscopy revealed viral particles in the feces of *D. innubila* similar in fine structure to other described Nudiviruses.

DiNV is associated with greatly reduced survival and offspring production among wild-caught individuals of *D. innubila* and with greatly reduced offspring production in *D. falleni*. While this association does not prove that these viruses cause the reduced fitness, the data strongly suggest that DiNV is a highly pathogenic infection. Furthermore, the prevalence of DiNV infection in natural populations of *D. innubila* from the Chiricahua Mountains of Arizona was consistently high, around 40%, in 2 successive years, suggesting that this virus may cause a major reduction in mean absolute fitness within this species. The prevalence of infection was similarly high in other members of the quinaria group – *D. munda* and *D. tenebrosa* – in collections from the Chiricahua Mountains. The prevalence of infection within *D. falleni*, a quinaria group species common in the eastern North America, was much lower, around 3%. The frequency of infection in species outside the quinaria group was more sporadic. Darren Obbard has found a similar virus in the melanogaster group, but his nucleotide sequences are about 25% diverged from those found in this study (personal communication).

Microinjection of DiNV-positive filtrate into lab-reared flies further suggests that the virus is highly pathogenic. Most flies injected with the virus died within two weeks of injection. In both species, males survived longer than females, and in both sexes, *D. falleni* survived longer than *D. innubila*. The difference between species may be due to size differences in flies (*D. falleni* used in the experiment were larger than *D. innubila* and may therefore require a higher virus titer for the same pathogenic effect), or could reflect selection for increased virulence of the virus to its natural host, since the virus injected was from *D. innubila*. The difference between the sexes cannot be explained by size since males are smaller and survived longer. Interestingly, overall and in the 2010 *D. innubila* collection, males had higher rates of infection than females (see Table 4). This is consistent with the lower mortality in males observed in the lab.

Vertical transmission of DiNV is unlikely to be important in the population, since the rate of transmission from mothers to offspring in the laboratory culture was <5% in *D. innubila*. The single instance of mother-offspring transmission may have mediated via a fecal-oral route, as this is the predominant mode of transmission in OrNV [41]. Further supporting fecal-oral transmission, virus genes were PCR-amplified and virus particles were found using electron microscopy in fly fecal material. Thus, viral infections in natural populations may result from horizontal transmission among adult flies and their offspring at their mushroom feeding and breeding sites.

Phylogenetic analysis of the partial viral P47 shows that the DiNV infecting *D. innubila* and *D. falleni* form closely related but genetically distinct clades. Thus, DiNV is a geographically widespread, prevalent, and pathogenic DNA virus for which members of the Drosophila quinaria group appear to be particularly important hosts.

### A new protocol for virus discovery

One goal of the metagenomics survey was to show that the protocol could detect virus in a single fly. In a 40-fly sample spiked with a single fly infected with DCV, 2/3 of all reads had DCV as a best hit, demonstrating a high level of sensitivity of the protocol. The almost complete absence of such hits in the sample not spiked with DCV attests to the specificity of the method. In addition to DCV, our screen uncovered several putative viral sequences. The most abundant of these were assigned to DiNV.

Given the success in recovering DCV from the spiked sample, why weren't more viruses found? Most sequences found some hits, so although there could be some virus sequences in our dataset with no homologs in the Refseq database, most sequences were readily assignable. There are at least 3 other possibilities. First, viral capsids may vary in their ability to protect viral RNA from degradation by RNAse, allowing some viruses to go undetected. Second, some viruses may be rare in *D. innubila*. Given that *Wolbachia* may provide some protection against RNA virus infection, the prevalence of RNA virus infection may be driven down by *Wolbachia* in *D innubila*, making them harder to detect. Finally, rare and virulent viruses may not have been present in the sample of 80 flies used, since infection frequency and virulence are usually assumed to be negatively correlated.

Most well-studied viruses of *Drosophila* have minor fitness effects in flies that either inherit the virus vertically or contract it through feeding, but greater effects when flies are injected with the virus [28]. This lack of virulence is perhaps because those viruses that are well-studied were discovered in cell culture or laboratory stocks and are therefore by nature less virulent since stocks and cell lines with very virulent viruses would not last long. This ascertainment bias is lessened in surveys of natural populations since captured flies could be quite sick. While we do not know the natural route of infection for any *Drosophila* viruses (although some are at least partly vertically transmitted), it is probably safe to conclude that DiNV is not vertically transmitted and infection frequencies are high enough that infection via mites (which are found at relatively low frequency on *D. innubila*), the natural analog of microinjection, is unlikely. Therefore, DiNV appears to be a virus exhibiting high virulence without requiring a rather drastic injection to show such effects.

The discovery of a DNA virus that naturally infects *Drosophila* opens the way for study of host immune response to DNA virus infection in an easily cultured species. The system also lends itself to studies of host-pathogen coevolution between geographically isolated sister species and between semi-isolated Sky Island populations of *D. innubila*.

## Supporting Information

Material S1 Sequences for short DiNV orthologs of other Nudiviruses. Note these are too short (<200bp) to be published in Genbank.
(DOC)

Figure S1 Schematic of viral nucleic acid enrichment protocol. Note there is no DNA digestion during the RNA extraction.
(EPS)

Figure S2 Distribution of BLAST hits corresponding to Drosophila C virus according to position in the genome.
(PNG)

Figure S3 The phylogenetic relationships of DiNV isolated from *Drosophila* species collected near Portal, AZ (blue) and Rochester, NY (red). Branch labels are posterior probability/maximum likelihood bootstrap support.
(PDF)
