# Supplementary material for: A DNA Virus of Drosophila
Source: PLoS One. 2011 Oct 28;6(10):e26564. doi: 10.1371/journal.pone.0026564 (PMC3203887; doi:10.1371/journal.pone.0026564)
Supplement: Material S1 — Sequences for short DiNV orthologs of other Nudiviruses. Note these are too short (<200bp) to be published in Genbank. (DOC) [file pone.0026564.s001.doc]

**SUPPLEMENTAL ONLINE MATERIAL S1**

>Drosophila innubila Nudivirus polh/gran-like protein, partial sequence

acttcttctggtatacggccaatggaagtgccaatattcattaaagtgttatgcaaataatcgtatgatgttactgcaatagcttcttcaacgttatgtttggttaaattcgatatactaggtgt

>Drosophila innubila Nudivirus polh/gran-like protein, partial sequence (protein)

NLTKHNVEEAIAVTSYDYLHNTLMNIGTSIGRIPEE

>Drosophila innubila Nudivirus vp91-like protein, partial sequence.

aatagctggcatcattaagtttaattgtatcgaaaccacgattaaagcatggtgttccagttatgcaaccgccaacgtcattgctatattttgtatcattggcgcacgtttgtaatacgctacgattattttcacacatgtaaaattcatttgtattc

>Drosophila innubila Nudivirus vp91-like protein, partial sequence (protein)

TNEFYMCENNRSVLQTCANDTKYSNDVGGCITGTPCFNRGFDTIKLNDASY

>Drosophila innubila Nudivirus GrBNV gp72-like protein, partial sequence

gcacaatgacattgtgttggatgattacgtttatgttgcaatgaattggatattattgccttgaaatatttaaacagttgcattttcggaccatacagaaccaaaataatatcaactttacataattggttgccatctgcgacatttcaatatgccacagtttcgatacaacgacaaggcacacaggggatagg

>Drosophila innubila Nudivirus GrBNV gp72-like protein, partial sequence (protein)

LRLCCNELDIIALKYLNSCIFGPYRTKIISTLHNWLPSATFQYATVSIQRQGT

>Drosophila innubila Nudivirus GrBNV gp78-like protein, partial sequence

CAAAAGCCAATGGATTGAAGAAATCATTTGTACGATTTATAATCGTAGGAGTTAGTTTTCCATAGCACGATTCATAAAATGGTTGATATTGATAGTTCATGAAAACATCCAAAGCGTCCCCACGACGAGCATTATAATATACTCGAGATGGTTTATGAATGTGTATCATTTCAG

>Drosophila innubila Nudivirus GrBNV gp78-like protein, partial sequence (protein)

EMIHIHKPSRVYYNARRGDALDVFMNYQYQPFYESCYGKLTPTIINRTNDFFNPLAF
