# Supplementary figures and images for: A DNA Virus of Drosophila
Source: PLoS One. 2011 Oct 28;6(10):e26564. doi: 10.1371/journal.pone.0026564 (PMC3203887; doi:10.1371/journal.pone.0026564)

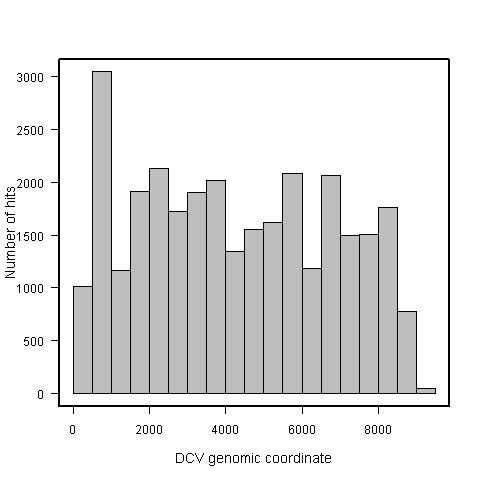

Supplement: Figure S2 — Distribution of BLAST hits corresponding to Drosophila C virus according to position in the genome. (PNG) [file pone.0026564.s003.png]

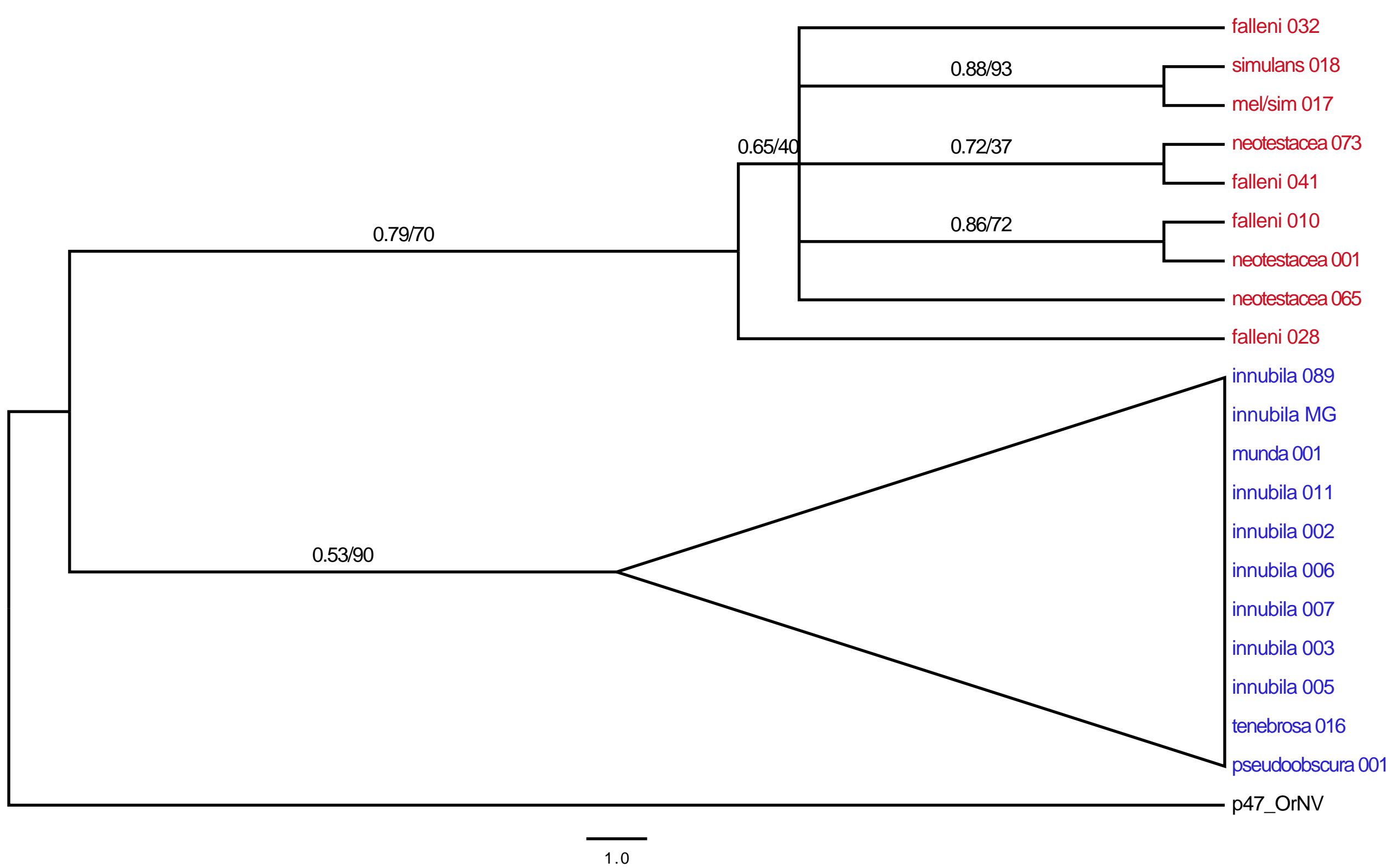

Supplement: Figure S3 — The phylogenetic relationships of DiNV isolated from Drosophila species collected near Portal, AZ (blue) and Rochester, NY (red). Branch labels are posterior probability/maximum likelihood bootstrap support. (PDF) [file pone.0026564.s004.pdf]
